# Mobile HIV Screening in Cape Town, South Africa: Clinical Impact, Cost and Cost-Effectiveness

**DOI:** 10.1371/journal.pone.0085197

**Published:** 2014-01-22

**Authors:** Ingrid V. Bassett, Darshini Govindasamy, Alison S. Erlwanger, Emily P. Hyle, Katharina Kranzer, Nienke van Schaik, Farzad Noubary, A. David Paltiel, Robin Wood, Rochelle P. Walensky, Elena Losina, Linda-Gail Bekker, Kenneth A. Freedberg

**Affiliations:** 1 Division of Infectious Disease, Massachusetts General Hospital, Boston, Massachusetts, United States of America; 2 Division of General Medicine, Massachusetts General Hospital, Boston, Massachusetts, United States of America; 3 Medical Practice Evaluation Center, Massachusetts General Hospital, Boston, Massachusetts, United States of America; 4 Harvard University Center for AIDS Research (CFAR), Boston, Massachusetts, United States of America; 5 Desmond Tutu HIV Centre, Institute of Infectious Disease and Molecular Medicine, Faculty of Health Sciences, University of Cape Town, Cape Town, South Africa; 6 Department of Infectious Disease Epidemiology, London School of Hygiene and Tropical Medicine, London, United Kingdom; 7 The Institute for Clinical Research and Health Policy Studies, Tufts Medical Center, Boston, Massachusetts, United States of America; 8 Tufts Clinical and Translational Science Institute, Tufts University, Boston, Massachusetts, United States of America; 9 Department of Health Policy and Management, Yale School of Public Health, New Haven, Connecticut, United States of America; 10 Department of Medicine, Faculty of Health Sciences, University of Cape Town, Cape Town, South Africa; 11 Division of Infectious Disease, Brigham and Women's Hospital, Boston, Massachusetts, United States of America; 12 Departments of Biostatistics and Epidemiology, Boston University School of Public Health, Boston, Massachusetts, United States of America; 13 Department of Orthopedic Surgery, Brigham and Women's Hospital, Boston, Massachusetts, United States of America; 14 Department of Health Policy and Management, Harvard School of Public Health, Boston, Massachusetts, United States of America; University of Missouri-Kansas City, United States of America

## Abstract

**Background:**

Mobile HIV screening may facilitate early HIV diagnosis. Our objective was to examine the cost-effectiveness of adding a mobile screening unit to current medical facility-based HIV testing in Cape Town, South Africa.

**Methods and Findings:**

We used the Cost Effectiveness of Preventing AIDS Complications International (CEPAC-I) computer simulation model to evaluate two HIV screening strategies in Cape Town: 1) medical facility-based testing (the current standard of care) and 2) addition of a mobile HIV-testing unit intervention in the same community. Baseline input parameters were derived from a Cape Town-based mobile unit that tested 18,870 individuals over 2 years: prevalence of previously undiagnosed HIV (6.6%), mean CD4 count at diagnosis (males 423/µL, females 516/µL), CD4 count-dependent linkage to care rates (males 31%–58%, females 49%–58%), mobile unit intervention cost (includes acquisition, operation and HIV test costs, $29.30 per negative result and $31.30 per positive result). We conducted extensive sensitivity analyses to evaluate input uncertainty. Model outcomes included site of HIV diagnosis, life expectancy, medical costs, and the incremental cost-effectiveness ratio (ICER) of the intervention compared to medical facility-based testing. We considered the intervention to be “very cost-effective” when the ICER was less than South Africa's annual *per capita* Gross Domestic Product (GDP) ($8,200 in 2012). We projected that, with medical facility-based testing, the discounted (undiscounted) HIV-infected population life expectancy was 132.2 (197.7) months; this increased to 140.7 (211.7) months with the addition of the mobile unit. The ICER for the mobile unit was $2,400/year of life saved (YLS). Results were most sensitive to the previously undiagnosed HIV prevalence, linkage to care rates, and frequency of HIV testing at medical facilities.

**Conclusion:**

The addition of mobile HIV screening to current testing programs can improve survival and be very cost-effective in South Africa and other resource-limited settings, and should be a priority.

## Introduction

An estimated 6 million people in South Africa are living with HIV/AIDS, and 300,000 die annually of their disease [1]. Access to timely and effective antiretroviral therapy (ART) can dramatically reduce HIV/AIDS-related morbidity and mortality and decrease HIV transmission [2]–[8]. The South African government has committed to increase the number of people on ART by expanding treatment initiation criteria to include CD4 counts ≤350/µL [9], [10]. To realize this goal, a national HIV Counseling and Testing (HCT) campaign started in 2010 with the objective to test 15 million people by 2011 and continues to scale-up services to provide annual HIV testing for everyone in South Africa in upcoming years [11], [12]. This requires novel approaches to HIV screening, particularly in populations that are difficult to access through conventional strategies.

HIV counseling and testing on mobile units has begun to be implemented throughout sub-Saharan Africa and has been targeted by the South African government as a strategy to introduce HCT services directly into communities [12]–[18]. Mobile units can access first-time testers [14], [15], hard-to-reach populations (such as men [14], [18] and rural populations [14]–[16]) and individuals at high risk for HIV infection [13], [16]. In addition, mobile units diagnose individuals with HIV at higher CD4 counts than medical facility-based testing [18]. Our objective was to examine the value of mobile unit HIV testing in Cape Town, South Africa.

## Methods

### Analytic Overview

We use a widely-published HIV disease simulation model, the Cost-Effectiveness of Preventing AIDS Complications International (CEPAC-I) model, to evaluate the clinical and economic value of adding a mobile HIV testing unit, including point-of-care (POC) CD4 count testing, to current medical facility-based HIV testing in Cape Town [19]–[25]. We project sites of HIV diagnosis and linkage to care, life-expectancy, 5-year survival and engagement in care, and HIV-related medical care costs for a population offered HIV testing by one of two strategies: 1) medical facility-based HIV testing, in which outpatients undergo diagnostic testing via a primary health care clinic's pre-existing HCT program; or 2) the mobile unit intervention as well as medical facility-based testing (hereafter referred to as the mobile unit intervention), the addition of a one-time offer of an HIV test and POC CD4 count via a mobile testing unit for those HIV-infected. Both strategies are simulated in the same community; undiagnosed HIV-infected patients can also link to clinical care following presentation with an AIDS-defining opportunistic infection (OI). Cohort characteristics are derived from a study of patients tested in a mobile unit in Cape Town, South Africa from March 2010–September 2011 [26].

HIV-related costs are assessed from a modified societal perspective (excluding patient travel time and lost wages) and are reported in 2012 US$. We report all outcomes used to inform resource allocation decisions on a present-value basis using a 3% discount rate [27]. Comparative value is expressed using an incremental cost-effectiveness ratio (ICER) to compare strategies calculated as the additional discounted cost of the mobile unit intervention divided by its additional benefit. Guided by the recommendations of the WHO [28], we define an intervention as “very cost-effective” when its ICER is less than South Africa's annual *per capita* Gross Domestic Product (GDP) ($8,200 in 2012), and “cost-effective” if less than three times the South Africa annual *per capita* GDP [28], [29]. We conduct sensitivity analyses by varying major input parameters to evaluate input uncertainty on cost-effectiveness results. We also calculate the total undiscounted HIV-related costs for the cohort over the initial 2 years to evaluate the budgetary impact of the intervention.

### The Cost-effectiveness of Preventing AIDS Complications International (CEPAC-I) model

We use the CEPAC-I model, a validated state transition computer model, to simulate natural history, screening, engagement in care, clinical management and costs of HIV disease. We use both the CEPAC-I Screening and Disease Models (Appendix S1), which have been described in detail previously [21], [30], [31]. The Screening Model determines whether and when HIV-infected individuals are diagnosed and link to care. Only after HIV-infected patients are diagnosed and successfully link to care will they be eligible for HIV treatment. The Disease Model assesses each simulated patient's clinical progression and treatment; patient engagement in care is defined by whether patients are in care, become lost to follow-up, and/or return to care.

#### Screening Model

We simulate HIV screening at the population level and account for both HIV-infected and HIV-negative individuals. HIV testing and linkage to care occur in one of three ways: 1) following presentation with an AIDS-defining OI (Appendix S1); 2) via the medical facility-based program; or 3) via the mobile unit intervention. To bias the analysis in favor of medical facility-based testing, HIV detection by the medical facility-based program or in the setting of an acute OI is assumed to be 100% sensitive and results in successful linkage to care. For simulated individuals in the mobile unit intervention, we vary the probabilities of accepting an HIV test, completing a POC CD4 count, and subsequently linking to care via the mobile unit.

#### Disease Model

Individuals in both screening strategies experience the same HIV disease progression, response to treatment, engagement with care (after initial linkage) and mortality. In the Disease Model, all HIV-infected individuals undergo monthly transitions between health states that depend on CD4 count, HIV RNA, and incidence/history of acute OI infection. Declines in CD4 count increase the risk of OIs and HIV-related mortality. After diagnosis and linkage to care, patients receive guideline-concordant care with CD4 count and HIV RNA monitoring, and are eligible for prophylaxis and ART [9], [32]. Those in care have a monthly probability of becoming lost to follow-up (LTFU) both before and after they initiate ART. Those LTFU have a probability of returning to care in the month of an acute OI infection, and a monthly probability of return to care after their first year lost.

### Input Parameters

#### Baseline Cohort Characteristics

To characterize the simulated cohort for both the medical facility and mobile unit testing strategies, we use population and clinical data, as well as health care utilization and cost data from a mobile testing unit deployed in Cape Town, South Africa [26]. When data from the mobile unit are not available, we use data from South African studies, including the Cape Town AIDS Cohort (Table 1) [33]–[41]. Males and females are analyzed separately to account for statistically significant sex-based differences in the likelihood of accepting a mobile unit HIV test, mean CD4 count at diagnosis, and subsequent linkage to care among individuals tested at the mobile unit with a CD4 count >350/µL (all p<0.05). Outcomes are weighted based on the sex distribution of people testing at the mobile unit (44% male). Mean mobile unit POC CD4 count at diagnosis for males is 423±236/µL and for females is 516±272/µL. The mean medical facility-based laboratory CD4 count at diagnosis is lower than the mean mobile unit CD4 count, at 291±203/µL for males and 357±242 for females [18], [26]. The prevalence of undiagnosed HIV among medical facility and mobile unit testers is 6.6% [26].

**Table 1:** Summary of base case input parameters and sensitivity analyses ranges examined for an analysis of a mobile HIV testing unit Cape Town, South Africa.

Variable		Base Case	Range	Ref.
**Baseline cohort characteristics**				
Male subjects (%)		44	30–80	[26]
Age, mean years (SD)		33 (13)	20–44	[26]
Prevalence of undiagnosed HIV (%)		6.6	1–30	[26]
HIV-infected patients CD4 count at diagnosis (mean cells/µL (SD))				
Mobile unit testing	Male*	423 (236)		[26]
	Female	516 (272)		[26]
Medical facility-based testing†	Male	291 (203)		[18], [26]
	Female	357 (242)		[18], [26]
**HIV screening**				
Mobile unit testing characteristics (one-time HIV test and POC CD4 count offer)				
HIV test acceptance probability (%)	Male*	97	70–99	[26]
	Female	95	70–99	[26]
Initial HIV test (Bioline HIV-1/2 3.0, Standard Diagnostics, South Korea)				[57]
Test sensitivity (%)		100		
Test specificity† (%)		99		
Confirmatory HIV test (Determine HIV-1/2, Abbott Laboratories, UK)				[57]
Test sensitivity (%)		100		
Test specificity† (%)		100		
CD4 count POC test (Alere PIMA™ Analyzer, Waltham, MA, USA)				
CD4 count acceptance probability (%)		91	70–99	[26]
CD4 >350 cells/µL	Male*	31	20–98	
	Female	51	20–98	
CD4 201–350 cells/µL		49	20–98	
CD4 <200 cells/µL		58	20–98	
Medical facility-based program average HIV test frequency				[18] †
HIV positive result		Every 4.0 yrs	1–10 yrs	
HIV negative result		Every 5.7 yrs	1–10 yrs	
**Loss to follow-up and return to care**				
Probability of loss to follow-up				[58], [59] †
Pre-ART (monthly)		0.0108	0.005–0.02	
On ART (monthly)	Adherence >95%	0.0016		
	Adherence <50%	0.0108	0.005–0.02	
Probability of return to care				Assumption
With acute WHO stage 3–4 disease or TB		0.50		
Without WHO stage 3–4 disease or TB after first year lost (monthly)		0.01	0.005–0.02	
**ART treatment**				
Initiation at WHO stage 3–4 disease presentation, TB, or CD4 <350/µL				[9], [32]
Monthly CD4 count increase on suppressive ART (cells/µL)				[41]
Initial 8 weeks		67		
After 8 weeks		3		
**HIV screening costs (2012 US$)**				
Mobile testing intervention (2-year) (×1,000)				[26]
Purchase and modification		152.0		
Mobile van resale value		(56.4)		
Medical/counselor salaries‡		216.2		
Administrative salaries/maintenance§		209.8		
Total 2-year mobile unit intervention cost∥		521.6	250–1,000	
No. of individuals offered a test over 2-yrs		18,870	9,440–28,310	
Per-person mobile unit cost (excluding HIV test costs)		27.60	13.60–54.40	
Initial HIV test		1.70	0–8.50	
Confirmatory HIV test		2.00		
Total per-person mobile unit cost (including HIV test costs)				
HIV-negative result		29.30	14.70–44.00	
HIV-positive result		31.30	15.70–46.95	
POC CD4 count		7.70	0–38.00	
Medical facility-based HIV testing programs¶				[45]
HIV-positive result		13.90		
HIV-negative result		9.30		
**Clinical care costs (2012 US$)**				
Co-trimoxazole prophylaxis cost (monthly)		1.40		[34]
ART regimen cost (monthly)				[33]
First-line		13.30		
Second-line		40.30		
Laboratory CD4 count test cost		13.90		[35]
HIV RNA cost		69.50		[35]
Inpatient hospital cost, per day		315.10		[35]
Outpatient hospital cost, per visit		32.60		[35]

* Statistically significant difference between males and females.

† Parameter derived and/or calculated from reference data.

‡ Comprising of one nurse practitioner ($84,200), one registered nurse ($71,100), three counselors ($47,500), one educator ($1,200) and one nurse practitioner at 20% time ($12,200).

§ Made up of one driver ($27,100), one project manager ($152,300), one data capturer/administrator ($22,000), diesel ($7,500) and general maintenance ($900).

∥ Assumed that van could be resold after 2 years of use.

¶ Costs include initial and confirmatory HIV test, staff salaries, and space in a voluntary counseling and testing site.

SD: standard deviation; POC: point-of-care; ART: antiretroviral therapy; WHO: World Health Organization; TB: tuberculosis.

#### Screening Characteristics

In the medical facility-based strategy, the average HIV testing frequency is once every 5.7 years among HIV-negative individuals and once every 4.0 years among HIV-infected individuals (Appendix S1) [18]. Following serial rapid HIV tests, newly diagnosed HIV-infected patients link to care and receive a laboratory CD4 count.

In the mobile unit intervention strategy, all individuals regardless of HIV status are offered a one-time rapid HIV test. The probability of accepting an HIV test, accepting a CD4 count, and linking to care are varied independently, and are not 100%. HIV test acceptance probabilities are derived from the likelihood of HIV test acceptance among individuals who enter the Cape Town mobile unit (males 97%, females 95%). Reactive results are confirmed by a second HIV rapid test. At the same visit, individuals with a positive HIV test are offered a POC CD4 count. Successful linkage to care is defined as a clinic visit within 3 months of mobile unit testing. Linkage to care is stratified by CD4 count and accounts for poorer linkage at higher CD4 counts. For individuals with an observed CD4 count ≤350/µL, linkage to care probabilities are stratified by observed CD4 count (49%–58%). For individuals with an observed CD4 count ≥350/µL, linkage to care probabilities are stratified by sex (males 31%, females 51%) because of significant differences noted by gender in the Cape Town mobile unit. In both males and females, linkage to care generally increases as observed CD4 count decreases, consistent with the literature [26], [42], [43]. Both HIV-infected individuals not diagnosed or linked via the mobile unit and HIV-negative individuals are eligible for repeat HIV testing as per the medical facility-based strategy throughout their lifetimes.

#### ART Treatment Characteristics

ART is initiated upon diagnosis of a severe AIDS-defining OI or TB, regardless of CD4 count, or with a CD4 count ≤350/µL [9], [44]. Depending on their ART adherence level, patients on ART experience a reduction in HIV RNA and a CD4 count increase. Two ART regimens are available to individuals over the course of their lifetime [32]. In the first 8 weeks of successful virologic suppression individuals experience a 67/µL CD4 count increase, followed by a 3/µL increase per month until virologic failure [41]. Individuals experiencing virologic suppression are susceptible to treatment failure resulting in virologic rebound and CD4 decline. Patients are switched from 1st to 2nd line ART after treatment failure is confirmed by an HIV RNA count showing a 10-fold increase in HIV RNA.

#### Costs

The cost to receive an HIV test in the medical facility-based program is $9.30 per negative result and $13.90 per positive result; these costs include the initial and confirmatory HIV tests, staff salaries and space in a voluntary counseling and testing site [45]. Clinic-based laboratory CD4 count cost is $13.90 [35].

Costs are calculated based on the Cape Town mobile unit's expenditure and utilization (Table 1) [26]. We allocate the cost of the mobile unit by dividing the total costs of acquisition, operation and maintenance (net of resale value) over its 2-year usable life ($521,600) by the estimated 18,870 persons who could be served in that period. This contributes an additional $27.60 to the startup cost for patients in the mobile unit intervention cohort. Each person also incurs medical supplies and waste disposal costs for an initial HIV test ($1.70) and a confirmatory test for reactive results ($2.00). This results in an estimated per-person cost of $29.30 per negative result and $31.30 per positive result. Diagnosed HIV-infected individuals also receive a POC CD4 count for $7.70.

On average, the Cape Town mobile unit operated 3 days per week, testing approximately 40 people per day, with fluctuations due to national holidays, weather, staffing, training and other activities. For the remaining 2 days of the work week, the mobile unit was deployed for an incentivized HIV testing program which is not included as part of this study. Assuming the mobile unit operates 5 days per week, at the capacity recorded from March 2010–March 2011, we determine that it could have tested 18,870 people over a 2-year intervention period [26].

All patients, regardless of where they were diagnosed, are subject to the same medical costs of HIV-related care. These costs include inpatient and outpatient services, laboratory tests, and ART regimens, for those eligible to be on ART.

#### Sensitivity Analyses

In sensitivity analyses, we vary multiple input parameters within plausible ranges to evaluate the impact on the cost-effectiveness results for mobile unit testing. Initially, we vary each major model parameter at a time, including the prevalence of undiagnosed HIV, mean age of testers, medical facility-based program average per-person HIV testing frequency, mobile unit HIV test and POC CD4 count acceptance probabilities, linkage to care probabilities, intervention cost, HIV test and POC CD4 count test costs, as well as the probability of LTFU and return to care. We then evaluate the interaction of the most influential parameters in multi-way sensitivity analyses.

#### Budget Impact Analysis

Based on widely-cited guidelines for the conduct of budget impact analysis [46], we evaluate the affordability of the intervention from the perspective of the Western Cape Department of Health, the entity that would be responsible for funding new HIV testing programs. For both strategies, we consider the undiscounted cost of mobile unit and medical facility-based HIV testing (including the 2-year capital cost for the mobile unit itself), ART and prophylaxis treatment, CD4 count and HIV RNA laboratory monitoring, and direct HIV-related inpatient and outpatient costs for the cohort over the first 2 years. Though costs do include inpatient and outpatient HIV care costs for both strategies, we do not account for any additional buildings or infrastructure that may be required to treat additional people identified in the mobile testing intervention. We do not take into account costs associated with lost time, lost productivity, or non HIV-related care, since these are not incurred by the Department of Health.

#### Ethics Statement

The study was approved by the University of Cape Town Health Sciences Faculty Ethics Committee (SA), and the Partners Health Care Human Research Committee (Protocol 2010P002636), Boston, Massachusetts, USA.

## Results

### Base Case

#### Life expectancy and cost-effectiveness

In the medical facility-based strategy, the discounted life expectancy was 249.9 months (or 20.8 years; undiscounted 449.7 months, or 37.5 years) in the overall population and 132.2 months (or 11.0 years; undiscounted 197.7 months, or 16.5 years) in the HIV-infected population (Table 2). The addition of a mobile unit intervention increased projected life expectancy by 0.5 months to 250.4 months (or 20.9 years; undiscounted by 1.0 month to 450.7 months, or 37.6 years) in the overall population and by 8.5 months to 140.7 months (or 11.7 years; undiscounted by 14.0 months to 211.7 months, or 17.6 years) for HIV-infected individuals. These results are comparable to other screening tests [47], [48]. Due to their higher CD4 count at detection, in both strategies females had a higher life expectancy than males. With the mobile unit intervention, females discounted life expectancy was 146.4 months compared to 133.4 for males.

**Table 2:** Model outcomes and cost-effectiveness of mobile unit HIV testing in Cape Town, South Africa.

	Medical facility-based testing	Mobile unit intervention
*Total population*		
Undiscounted life expectancy (months)	449.7	450.7
Discounted life expectancy (months)	249.9	250.4
Discounted per-person costs ($)	3,970	4,070
**Incremental cost-effectiveness ratio** * **($/YLS)**	**–**	**2,400**
*HIV-infected population*		
5-year survival (%)	69	73
Undiscounted life expectancy (months)	197.7	211.7
Discounted life expectancy (months)	132.2	140.7
Discounted per-person costs ($)	11,270	12,430
HIV RNA suppressed at 5 years (%)	35	39

* Incremental cost-effectiveness ratios <1× South African *per capita* gross domestic product ($8,200) considered very cost-effective based on WHO suggestions [28]. Costs in 2012 US$. Discounted at 3% per year (see methods). YLS: years of life saved.

The addition of the mobile unit intervention to current medical facility-based testing increased the discounted average per-person lifetime costs from $3,970 to $4,070, yielding an incremental cost-effectiveness ratio of $2,400/year of life saved (YLS) for the mobile unit intervention compared to medical facility-based testing.

#### Mechanisms of linkage to care

**Figure 1:**
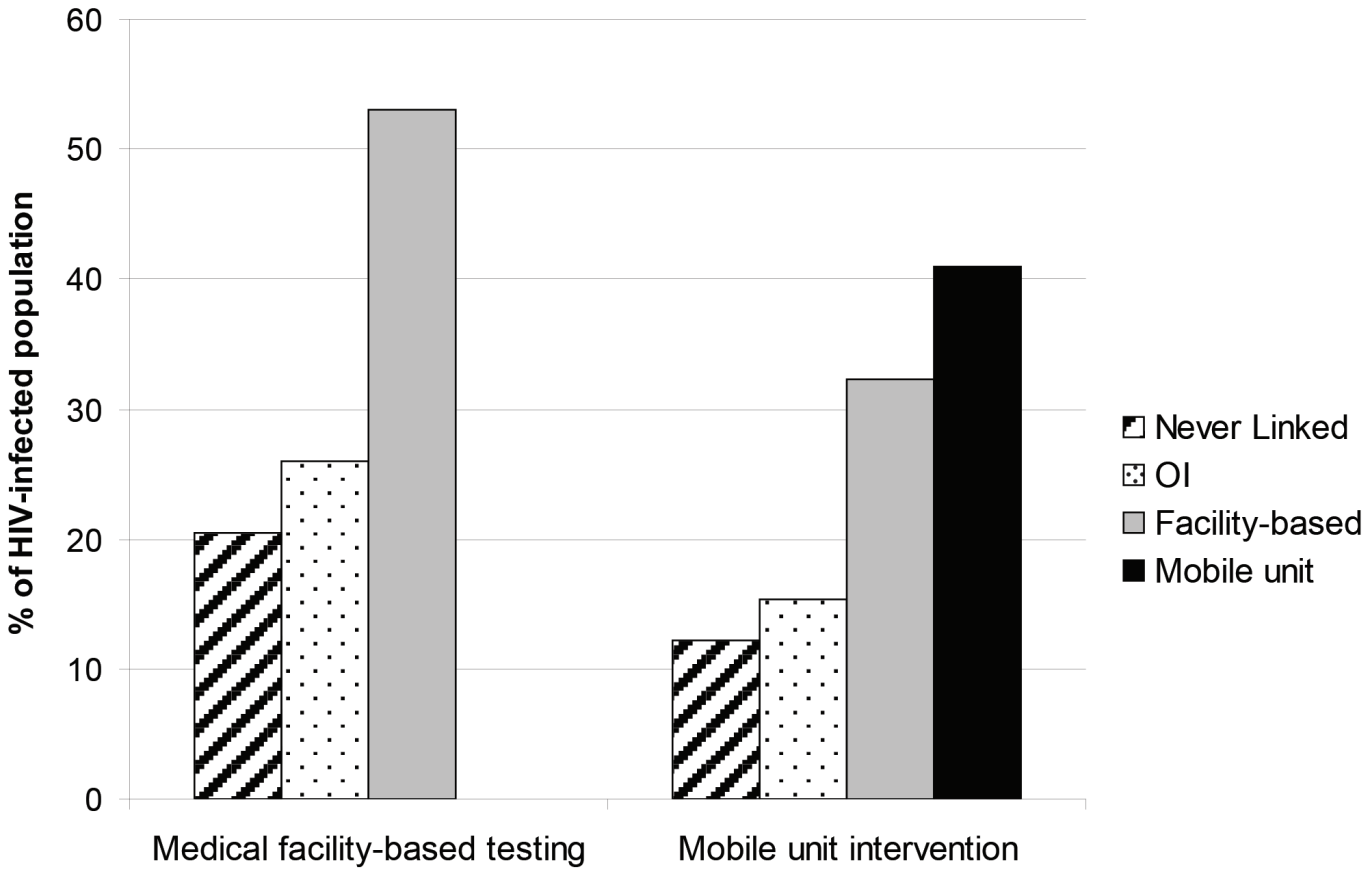
Model-derived mechanisms of HIV linkage in Cape Town, South Africa. The charts display the proportion of HIV-infected individuals linked to care with the medical facility-based strategy, and the mobile unit intervention strategy. HIV: human immunodeficiency virus, OI: Opportunistic Infection.

In the medical facility-based testing strategy, 21% of HIV-infected people died before linking to care and 26% were linked to care after they developed a severe AIDS-defining OI (including TB) (Figure 1). The remaining 53% were linked via medical facility-based testing. With the addition of the mobile unit intervention, 41% of HIV-infected people were linked to care via the mobile unit and 32% via medical facility-based testing. As a result, fewer people died without ever linking (12%) or after developing a severe AIDS-defining OI (including TB) (15%).

#### Survival and engagement in care at five years

**Figure 2:**
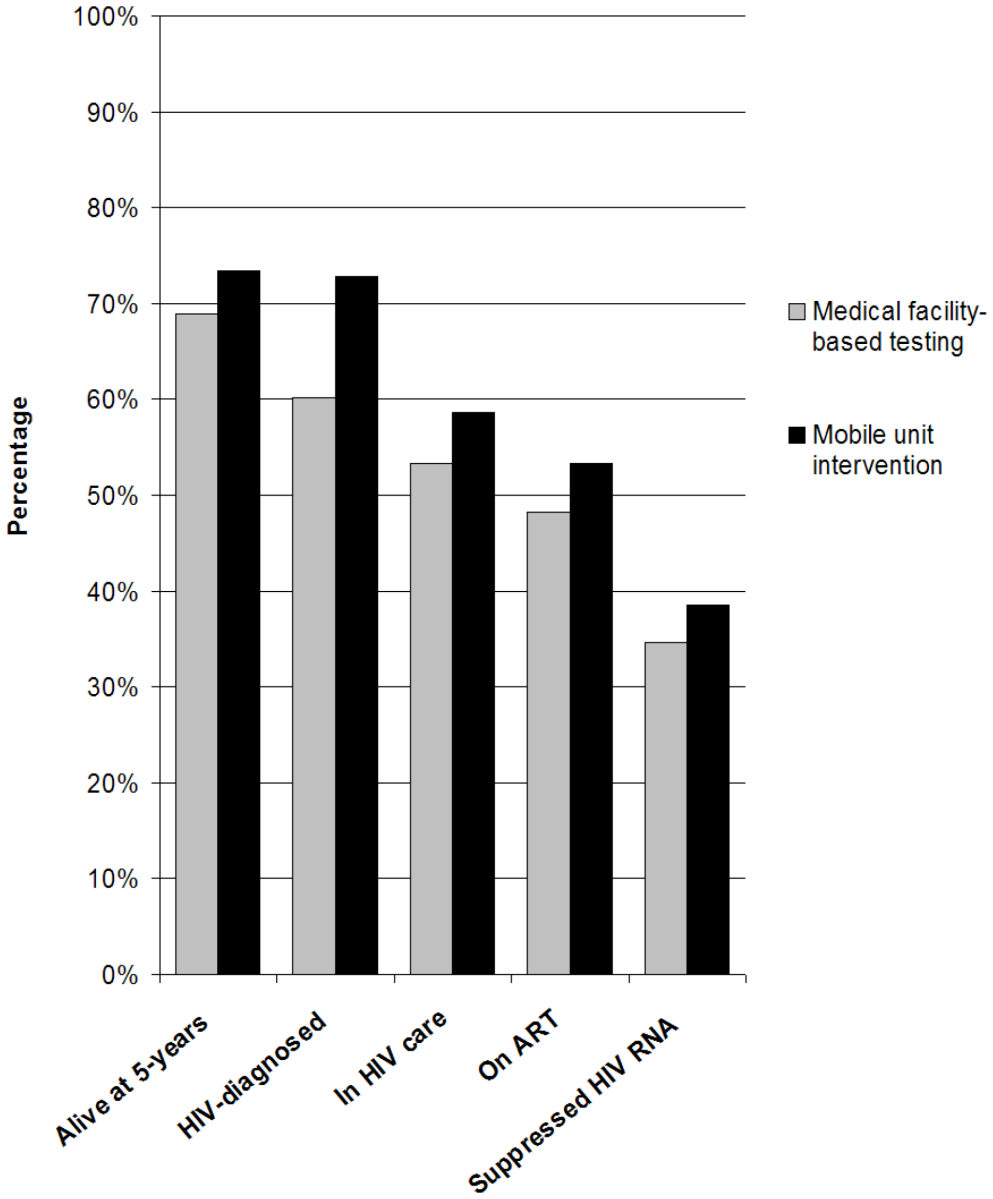
Model-derived survival and engagement in care of HIV-infected individuals in Cape Town at 5 years from the start of observation. The bar graphs shows the proportion (out of the initial 1,240 HIV-infected population) at 5 years who are alive, diagnosed, linked to and retained in care, are on ART, and are HIV RNA suppressed on ART. HIV: human immunodeficiency virus, RNA: ribonucleic acid, ART: antiretroviral therapy.

Five-year survival among the HIV-infected population in the medical facility-based testing strategy was 69% compared to 73% in the mobile unit intervention strategy (Figure 2). The mobile unit intervention was associated with more people on suppressive ART, 39%, compared to 35% with medical facility-based testing.

### Sensitivity Analyses

#### One-way sensitivity analyses

In one-way sensitivity analyses, we varied major input parameters independently within plausible ranges in both strategies (see Table 1 for ranges). The ICER for the addition of the mobile unit intervention remained <$2,800/YLS when we varied the mobile unit HIV test and POC CD4 count acceptance probabilities and costs, the mobile unit purchase and operation cost as well as LTFU and return to care (results in Appendix S1). The ICER of the mobile unit intervention was most sensitive to variations in the prevalence of undiagnosed HIV ($10,000/YLS when prevalence of undiagnosed HIV <0.5%) and linkage to care among mobile unit testers ($3,900/YLS if <20% linkage to care). The ICER was also sensitive to the medical facility-based program HIV testing frequency, but remained below $3,000/YLS even when the frequency of medical facility-based testing was once every year.

#### Multi-way sensitivity analyses

**Figure 3:**
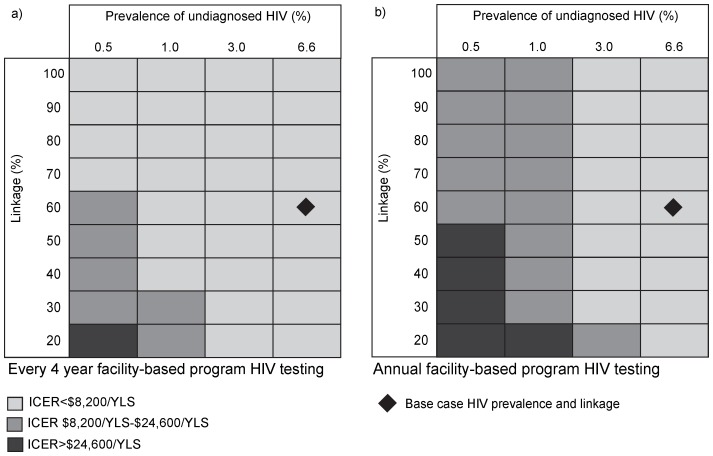
Multi-way sensitivity analyses on prevalence of HIV, linkage to care and facility-based HIV testing frequency. Prevalence of undiagnosed HIV is varied on the horizontal axis and linkage to care on the vertical axis. Figure a) represents the base case medical facility-based program HIV testing frequency of once every 4 years. Figure b) represents annual medical facility-based program HIV testing. Light gray represents scenarios with an incremental cost-effectiveness ratio (ICER) <1× South African *per capita* gross domestic product ($8,200), dark gray represents scenarios with an ICER $8,200/YLS to $24,600/YLS and black represents scenarios with an ICER >$24,600/YLS. HIV: human immunodeficiency virus, ICER: incremental cost-effectiveness ratio.

We used multi-way sensitivity analyses to evaluate the interplay of the most important input parameters identified in one-way sensitivity analyses: prevalence of undiagnosed HIV, linkage to care, and the medical facility-based testing program per-person testing frequency (Figure 3). With medical facility-based testing every 4 years, the mobile unit ICER remained <3× the annual *per capita* South African GDP unless the prevalence of undiagnosed HIV was below 0.5% and linkage to care was less than 70%. Even with annual HIV testing provided by the medical facility-based program, the addition of the mobile unit intervention to medical facility-based testing had an ICER <3× GDP unless the prevalence of undiagnosed HIV was below 1%.

#### Budget impact analysis

**Figure 4:**
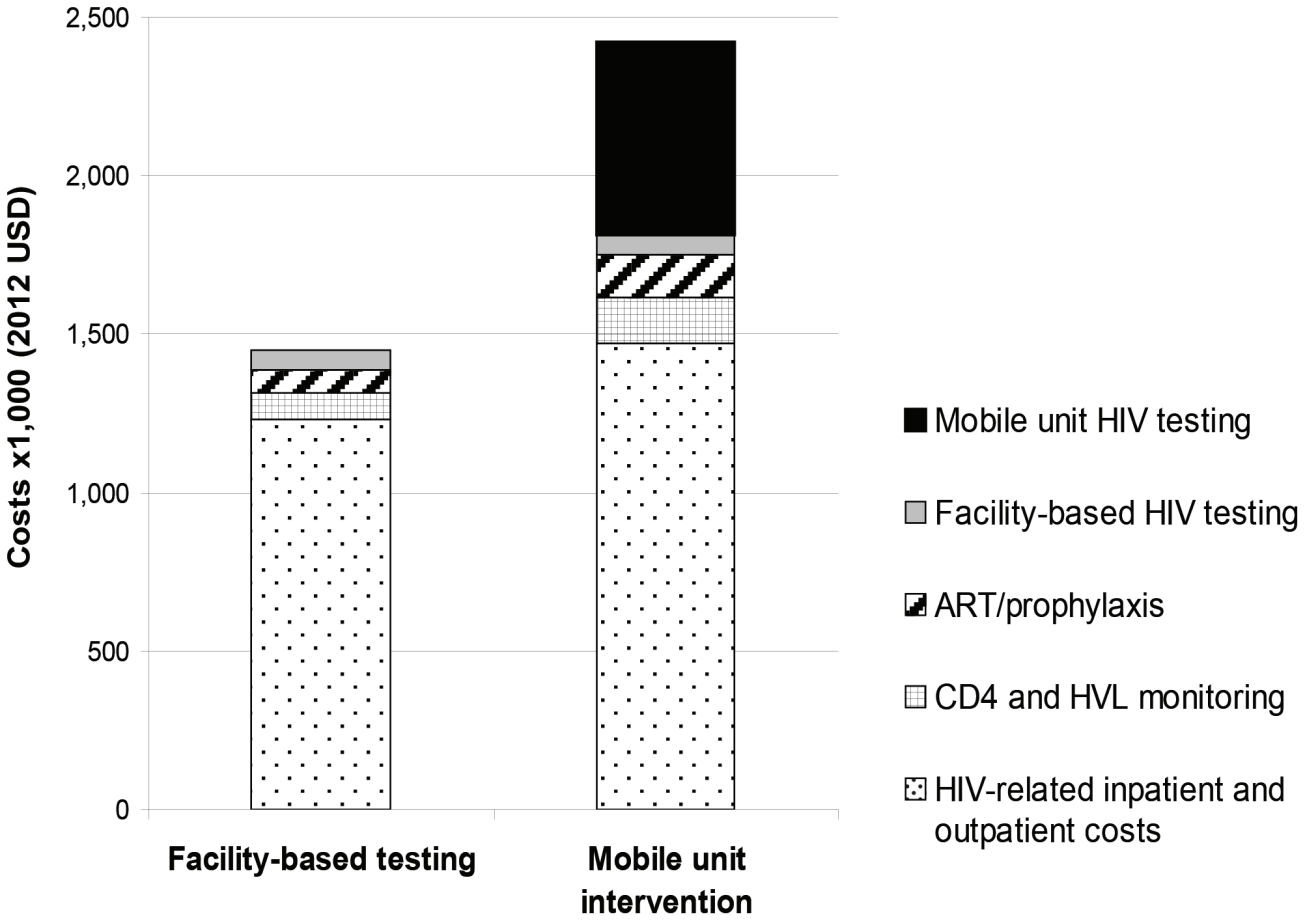
Total HIV-related cohort costs over initial 2 years in facility-based and mobile intervention strategies. This represents the total undiscounted costs for the cohort of 18,870 for HIV-related costs over the first 2 years, to be incurred by the Western Cape Department of Health. For both strategies HIV-related costs are comprised of HIV screening, routine CD4 and HIV RNA monitoring, ART and prophylaxis, and HIV-related inpatient and outpatient costs. Costs are ×1,000 USD. HIV: human immunodeficiency virus, ART: antiretroviral therapy.

For the 18,870 people projected to be evaluated by the mobile unit over 2 years (of whom 1,240 are HIV-infected), the total undiscounted HIV-related costs over the 2 years increased by $900,000 (from $1.5 million to $2.4 million), an increase of 67% compared to the facility-based testing strategy (Figure 4). Roughly $300,000 of this was attributable to the increased costs of care for the 190 additional cases identified and linked. The other $600,000 represents the costs of the mobile screening program.

## Discussion

The successes of mobile units in sub-Saharan Africa demonstrate the potential for major benefit with mobile unit HIV testing and integrated staging by POC CD4 in South Africa and other resource-limited settings [13], [15], [17], [18], [26]. We used data from a Cape Town mobile HIV testing unit, as well as the costs of testing and treatment, in a validated HIV simulation model to assess the clinical impact, cost, cost-effectiveness and the budgetary impact of a mobile HIV testing intervention. We found that the mobile unit intervention would increase the discounted life expectancy of HIV-infected individuals in a prevalent cohort in Cape Town by 8.5 months and increase the proportion that both link to care and initiate ART. We found that, at a 5-year horizon from the start of observation, the proportion of HIV-infected people alive in care increased from 53% to 59% and the proportion with suppressed viral loads increased from 35% to 39%. This increase would result in many lives saved, particularly in a region that has a high prevalence of HIV, such as many areas in sub-Saharan Africa. Further, because we used different cohort parameters, this underestimates the actual impact of the intervention by not accounting for the substantial proportion of patients in the medical facility intervention who died from opportunistic infections and other AIDS-related complications prior to HIV diagnosis. Despite start-up costs for the mobile unit totaling $152,300, the incremental cost-effectiveness ratio of the addition of the mobile unit was $2,400/YLS when compared to standard medical facility-based testing alone because the prevalence of undiagnosed HIV was so high in the population. This ICER is considerably less than the annual *per capita* GDP of South Africa, which we considered to be very cost-effective [28].

The mobile unit intervention remained very cost-effective in nearly all sensitivity analyses and was particularly robust to changes in the mobile unit costs including intervention start-up, operation, maintenance, and laboratory tests. The mobile unit remained very cost-effective even if targeted to the lowest provincial prevalence of undiagnosed HIV in South Africa (Western Cape, 4.4% [49], [50]) as long as linkage to care probabilities were greater than 30%. These results suggest that even in settings with a lower yield of diagnoses and fewer individuals successfully linking to care, the benefits of the intervention were still well worth the financial investment. In Khayelitsha township located in greater Cape Town, the population is approximately 500,000 and the antenatal HIV prevalence is estimated to be as high as 31% [51]. If this area were targeted, substantially higher numbers of people would be identified and linked to care with mobile testing.

Mobile unit testing is likely to be added to, rather than replace, medical facility-based HIV screening programs, which will improve HIV detection and linkage to care. To be conservative, we conducted an incremental analysis and considered the mobile unit intervention in addition to facility-based testing, rather than as a substitute for facility-based testing. Even when medical facility-based HIV testing was as frequent as annually, the addition of mobile unit HIV testing in this high prevalence setting remained very cost-effective ($3,000/YLS). These results indicate that the potential benefits from mobile screening persist even with major improvements in medical facility-based testing, such as might be realized with antenatal clinic testing, and underscore how a combination of extensive medical facility-based with mobile unit HIV testing offers the potential for immense benefit in South Africa and elsewhere.

Mobile testing units facilitate HIV diagnosis prior to advanced disease [18], [26], [42]. In this analysis, because patients in the mobile unit are diagnosed earlier and have a higher CD4 count at diagnosis, fewer people linked to care after the development of a severe OI (15%) than when only medical facility-based testing was available (26%). There are substantial individual and public health benefits if people with HIV are diagnosed early, link to care promptly and initiate ART when eligible; these include decreased incidence of opportunistic infections with their associated morbidity and mortality, and fewer HIV transmissions [2]–[8]. Our results also showed more favorable outcomes among females than males, due to females' diagnosis at higher CD4 counts and their higher linkage to care probabilities.

Given the cost-effectiveness of the Cape Town mobile HIV testing unit, as well as the successes of a variety of programs within sub-Saharan Africa, mobile HIV testing units are clinically effective and feasible options in many resource-limited settings [13], [15]–[17], [52], [53]. Cost-effective interventions, however, are not necessarily inexpensive or affordable. Deploying a mobile HIV testing unit like the Cape Town-based mobile unit requires an upfront investment of approximately $600,000 and long-term additional care costs of approximately $300,000 for an additional 190 HIV cases identified and linked to care at 2 years. While this increase in cost may be too high for some resource-limited countries, the mobile unit model is very cost-effective by international standards and could be affordable in Cape Town and other similarly-resourced settings, particularly those with higher HIV prevalences. Assuming linear marginal returns to increased investment, if participation and costs are increased 10-fold, to account for expansion of services throughout the greater Cape Town area, the undiscounted cost for mobile HIV screening would be $2,766,200 per year for each of two years; this represents 8% of the Western Cape 2013–2015 Comprehensive HIV and AIDS grant [54]. If the prevalence of undiagnosed HIV of the Cape Town area in which the mobile unit was deployed remained constant at 6.6%, this would result in an additional 1,900 individuals in care at 2 years. It is possible that the mobile unit testing capacity is higher than seen in the current experience; increased numbers tested by each unit would make testing more cost-effective, as the start-up costs for the tester are amortized over larger numbers of clients. Even if the capacity of the unit was half that in the base case analysis (390 individuals tested monthly), the mobile unit ICER remained well below the *per capita* South African GDP ($3,000/YLS).

As the population in any one area is saturated with HIV testing, we anticipate a decrease in prevalence of undiagnosed HIV making marginal returns non-linear. A benefit, however, of mobile testing is that the yield (e.g. new HIV diagnoses) can be assessed in real time (e.g. on a monthly basis). As a result, regular deployment and re-deployment of a number of mobile units can be targeted to areas where the prevalence of new HIV diagnoses remains sufficiently high enough to make testing cost-effective, and mobile units could be moved to other areas with higher prevalence or eventually phased out of service.

Is mobile unit HIV testing economically feasible in sub-Saharan Africa? Mobile units have been deployed in urban and rural settings in multiple countries, including South Africa, Nigeria, Zimbabwe, Tanzania and Uganda [13], [15]–[17], [52], [53]. These mobile units have been utilized despite barriers to widespread implementation, such as the cost of acquisition, modification, operation and maintenance, particularly in highly resource-constrained settings. The diversity of approaches to using mobile units in the region demonstrates that the requirements for mobile testing unit implementation can be flexible to the needs and resources of health care providers and the target community. The Cape Town mobile unit in this analysis comprises of a van and trailer that underwent extensive modifications [26]; a different mobile unit deployed in rural South Africa instead consists of a truck and tents [14]. There are also variations in the workforce employed in different settings [13], [15]. While tradeoffs regarding capacity and quality of service should also be considered, tailoring the design and implementation of mobile units to different settings could further reduce costs.

There are several limitations to this analysis. First, we assumed that, with the exception of the mean CD4 count at the time of HIV diagnosis, the cohort characteristics and HIV disease progression were the same for the medical facility-based testing and the mobile unit intervention groups. This may, however, miss social and economic differences between the cohorts that could influence patients' ability to access and stay in care. Second, we based the prevalence of undiagnosed HIV for both the medical facility and mobile unit on data collected by the mobile unit. Though this may have underestimated the undiagnosed HIV prevalence at the medical facility as people often go to medical facilities when sick, we performed sensitivity analyses to explore uncertainty in this parameter. Third, we used data from multiple studies to define the cohort and in some cases were limited by availability of data. Although we stratified some input parameters by sex, we did not have sex-specific data on undiagnosed HIV prevalence, viral load, response to treatment, or AIDS-, OI- and toxicity-related mortality. While we anticipate that having sex-specific parameters might alter the results slightly, there is no reason to believe they would change policy conclusions. Fourth, we did not consider new HIV cases that would develop during the intervention, so only prevalent cases were considered over the 2-year period with the focus on one-time testing. Fifth, we did not account for the monetary benefits of HIV transmissions averted as a result of increased ART coverage with mobile HIV screening, nor did we quantify the benefits of transmissions likely averted due to risk reduction associated with HIV screening [55]. Lastly, we assumed that 100% of new diagnoses linked to care from the medical facility-based program and after presentation with an AIDS-defining OI, while mobile unit linkage to care was imperfect. These last three limitations all bias the analysis in favor of medical facility-based testing and against mobile unit testing. Therefore, our results should be interpreted as a lower-bound estimate of the full clinical impact and economic benefits of mobile testing.

The now well-established benefits of timely access to ART necessitate a strong commitment to effectively diagnose HIV-infected individuals and ensure that they link to and remain in care. In South Africa, the National Department of Health and the National Health Treasury have expressed the political and financial will to create more opportunities for frequent, regular HIV testing [10], [56]. This ambitious undertaking will only be feasible with high-yield, economically effective strategies. This analysis suggests that mobile HIV screening is very cost-effective in South Africa, substantially increases the life expectancy of HIV-infected individuals, and decreases morbidity compared to medical facility-based testing alone. While requiring up-front investment to get these mobile units on the road, mobile testing is an investment well worth making to achieve the combined aims of effective diagnosis, treatment and prevention of HIV disease in many resource-limited settings.

## Supporting Information

Appendix S1 **The technical appendix includes further details on the CEPAC model, model inputs and sources, and sensitivity analyses.**
(DOCX)

Figure S1 **One-way sensitivity analyses on the addition of mobile unit HIV testing to medical facility-based testing.** This tornado diagram summarizes the results of multiple 1-way sensitivity analyses on the incremental cost-effectiveness of the addition of mobile unit HIV testing to medical facility-based testing in Cape Town, South Africa. The horizontal bars represent the incremental cost-effectiveness ratio (ICER) range as a result of variations in each single model parameter. The solid vertical line indicates the base case ICER ($2,400/LYS). The dashed vertical line indicates the South Africa *per capita* gross domestic product (GDP, $8,200). YLS: years of life saved; POC: point of care. (range; base case); SOC: standard of care.
(TIFF)

Figure S2 **Two-way sensitivity analyses on mobile unit test acceptance and linkage to care.** This diagram shows the incremental cost-effectiveness of the addition of mobile unit HIV testing to medical facility-based testing under conditions of varied mobile unit test acceptance and linkage to care. Linkage to care is varied on the vertical axis and test acceptance is on the horizontal axis.
(TIF)
