# Supplementary material for: Mobile HIV Screening in Cape Town, South Africa: Clinical Impact, Cost and Cost-Effectiveness
Source: PLoS One. 2014 Jan 22;9(1):e85197. doi: 10.1371/journal.pone.0085197 (PMC3898963; doi:10.1371/journal.pone.0085197)
Supplement: Appendix S1 — The technical appendix includes further details on the CEPAC model, model inputs and sources, and sensitivity analyses. (DOCX) [file pone.0085197.s001.docx]

**Appendix S1**

**TECHNICAL APPENDIX**

**Mobile HIV screening in Cape Town, South Africa:**

**clinical impact, cost and cost-effectiveness**

Ingrid V. Bassett, MD, MPH, *et al.*

This work was supported in part by: the National Institute of Allergy and Infectious Disease: R01 AI058736; T32 AI007433 the Harvard University Center for AIDS Research P30 AI060354; the National Institute of Mental Health: R01 MH090326, R01 MH073445, the President’s Emergency Plan for AIDS Relief (PEPFAR); and the Claflin Distinguished Scholar Award. The content is solely the responsibility of the authors and does not necessarily represent the official views of the National Institutes of Health.

The mobile testing unit was partially funded by PEPFAR through the Anova Health Institute and the United States Agency for International Development program and received further funding through the Agence Française de Développement.

In areas where computer programming and methods are identical, the text from this Appendix is similar to the on-line Technical Appendix available from:

1. Walensky RP, Wood R, Fofana MO, Martinson NA, Losina E, et al. (2011) The clinical impact and cost-effectiveness of routine, voluntary HIV screening in South Africa. J Acquir Immune Defic Syndr 56: 26-35.

**INTRODUCTION** 4

**METHODS**

CEPAC Screening Model

Overview 5

Mechanisms of HIV detection and linkage to care 5

Medical facility-testing input derivations 8

CEPAC-I Disease Model

Overview 11

Natural history 12

Engagement with care 12

Prophylaxis and laboratory monitoring 14

ART treatment 14

**RESULTS**

Sensitivity analyses 15

HIV transmissions averted 18

**TABLES** 17

**FIGURE LEGENDS** 24

**INTRODUCTION**

The analysis described in the main manuscript is built upon the foundation of the Cost-Effectiveness of Preventing AIDS Complications (CEPAC) International Model, a computer simulation of the clinical management and economics of HIV disease. We have linked this disease simulation to a model of HIV screening, detection, and linkage-to-care. The technical details of both models have been described extensively in previous publications [1,2,3,4,5,6,7,8]. In this Technical Appendix, we provide methodological details and report input parameters that are not reported in the manuscript. We also report additional sensitivity analyses and results performed in the context of this manuscript.

**METHODS**

**CEPAC Screening Model**

***Overview***

The basic structure of the CEPAC Screening Model has been detailed previously [3,9]. In summary, the purpose of the Screening Model is to simulate the clinical and cost impact of HIV testing and linkage to care activities in a population of both HIV-infected and HIV negative individuals. We use data from a study on testers at a mobile HIV testing unit deployed in peri-urban Cape Town to define parameters of relevance, including: prevalence of previously undiagnosed HIV, mean age and CD4 count at diagnosis at mobile unit test, as well as HIV test attributes such as cost; likelihood of accepting a mobile unit HIV test; likelihood of subsequently accepting a point-of-care (POC) CD4 count and the likelihood of linkage-to-care following mobile unit POC CD4 count [10].

***Mechanisms of HIV detection and linkage to care***

In order to link to care and receive all of the benefits of HIV treatment, HIV-infected individuals must be detected and receive a CD4 count. HIV detection occurs following either an HIV test or diagnosis with a severe opportunistic infection (OI) indicative of HIV infection. Following detection, a patient must receive a CD4 count test and can successfully link to care. The observed CD4 count is used to determine the appropriate timing of treatment initiations based on South African treatment guidelines outlined in the input parameters section of this Technical Appendix.

In this analysis, HIV detection and linkage to care can occur via one of three discrete mechanisms:

1. Mobile unit intervention: In the mobile unit intervention strategy all individuals are offered a one-time rapid HIV test via the mobile unit and a point of care (POC) CD4 count in the same visit after which they can link to care. The probability of accepting an HIV test, accepting a CD4 count, and linking to care are varied independently. Upon linkage to care, patients receive a laboratory CD4 count and their observed CD4 count is used to determine their clinical treatment. If a person does not successfully link to care they continue to be eligible for linkage through the second and third mechanism. We model this screening program structure on the Cape-Town based mobile unit [10].
2. Medical facility-based: HIV-infected individuals in both strategies can receive an HIV-positive test result within the context of standard of care pre-existing medical facility-based HIV testing programs. Both undetected and previously detected (but not linked) individuals have a monthly probability of receiving this test over their entire lifetime. Linkage to care following facility-based testing is assumed to be 100%. Upon linkage to care, patients receive a laboratory CD4 count and their observed CD4 count is used to determine their clinical treatment. HIV-negative individuals in both strategies receive facility-based testing throughout their lifetime. We model this screening program structure on a primary health care clinic HIV counseling and testing program in the area that is serviced by the Cape Town-based mobile unit. Input derivations for facility-based testing are detailed on page 8.
3. WHO stage 3 and 4 OI or TB: Detection of HIV infection can take place because an infected individual seek medical care for a WHO stage 3/4 OI or TB. Both undetected and previously detected (but not linked) individuals are eligible to be detected and linked to care following presentation with an OI over their entire lifetime. Probability of detection via presentation with WHO stage 3 and 4 diseases was assumed to be 100%, and with TB is assumed to be 50% [11]. Of these diagnosed patients, 100% receive a laboratory CD4 count and link to care. Upon linkage to care patients receive a laboratory CD4 count and their observed CD4 count is used to determine their clinical treatment. The CD4 count stratified probabilities of an undiagnosed HIV-infected individual presenting to care with different OIs is taken from a study in Cape Town [12].

To bias the results against the mobile unit intervention, in the model we conservatively assume that detection via either medical facility-based testing or presentation with a WHO stage 3/4 OI or TB are under conditions of perfect HIV test accuracy, acceptance and linkage to care. However, in the mobile unit, likelihood of accepting tests and linkage to care are varied as detailed in the manuscript. In addition, a small proportion of HIV-infected individuals (0.09%) in this analysis are acutely HIV-infected at initialization [13,14,15]. We assume for the purpose of this analysis that the test specificity of the mobile unit rapid HIV test is zero during the 2 month “window” period.

To account for a decreased accuracy with POC CD4, the POC CD4 count results may vary from the “true” CD4 count with a defined % standard deviation [16]. While the clinical events within the model are predicated on model-generated “truth” regarding a patient’s CD4 count, the POC CD4 count is used to determine each patient’s probability of linkage to care. To account for the noted difference in linkage to care at different CD4 counts [10,17,18], linkage to care is varied by observed CD4 count stratum: (>500 cells/µl; 351–500 cells/µl; 201–350 cells/µl; 101–200 cells/µl; 51–100 cells/µl; and 0-50 cells/µl).

***Medical facility-based testing input derivations***

We calibrate our medical facility-based program testing frequency to the model-estimated testing frequency among testers diagnosed at a Clinic HIV counseling and testing center that operated in the same community as the Cape Town-based mobile unit [14]. The calibration is carried out in two steps: 1) we project the mean CD4 count of an undiagnosed South African population; 2) we project the per-person monthly probability of testing necessary for this population to be diagnosed at the mean CD4 count in the clinic and use this to define the HIV testing frequency among HIV-infected patients. We then use the testing frequency of HIV-infected individuals, along with data from a survey administered in the same Cape Town community, to calculate a testing frequency for HIV-negative individuals. Based on the model projected life expectancy of HIV-negative individuals, we then determine the mean number of medical facility-based tests an individual would receive over a lifetime and the corresponding cost.

**HIV-infected individuals’ HIV testing frequency**

1. Mean CD4 count of undiagnosed South Africa population

We simulate a cohort of acutely HIV-infected individuals and HIV-negative individuals susceptible to HIV-infection. At initialization, HIV prevalence is 10% and the incidence is 1.38% [14,19]. The mean CD4 count of acutely-infected individuals is 751±267/µL [20]. Patients who developed AIDS-defining OIs or TB (and would have been diagnosed following presentation) were removed from the cohort. We projected that after 20 years the population of undiagnosed HIV-infected individuals, with no access to HIV treatment or prophylaxis, had a CD4 count of 493±269/µL in males and 499±270/µL in females.

1. Testing frequency at medical facility-based program

To determine the per-person testing frequency we simulated an undiagnosed South African cohort that could only be diagnosed via medical facility-based testing. Patients who develop AIDS-defining OIs or TB (who are been diagnosed following presentation) are removed from the cohort. We calibrate the medical facility-based testing rate to match the mean CD4 count of patients diagnosed at a facility-based HIV counseling and testing (HCT) program serving the same area as the Cape Town mobile unit (291±203/μL for males and 357±242/μL for females) [21]. We do this by back calculating the testing frequency based on the CD4 count at diagnosis. This corresponds to a per-person HIV testing frequency of once every 4 years on average. We apply the monthly probability of testing to all HIV-infected individuals (both acute and chronic infection) in the model. Each HIV test cost $13.90, which includes the cost of the rapid HIV test kit, confirmatory HIV test, outpatient department counselor salaries and the cost of space [22]. Every HIV-infected patient incurs the test cost at the same time as their HIV and hospital-based test.

Although we only consider the clinic HCT program for this derivation, the Cape Town study from which we derive these data also report the mean CD4 count of patients diagnosed at a district hospital in the same area (231/μL for males and 359/μL for females) [21]. Because the CD4 count at diagnosis was lower in the hospital than in the clinic HCT program, calibrating to the hospital data results in a lower testing frequency (once every 45 years among males, and once every 5 years among females). To be conservative, we assumed that all HIV-infected individuals had the higher probability of testing derived from the clinic HCT program.

**HIV negative individuals HIV testing frequency**

In a self-report survey administered in Cape Town, 29% of adults had never received an HIV test [14]. Based on these results, we assume that 30% of HIV-negative individuals never receive an HIV test and the remaining 70% receive a test at the same frequency as the HIV-infected individuals. The weighted per-person testing frequency is once every 5.7 years. In this analysis, the projected HIV-negative population life expectancy (beginning at age 33 years) is 35.7 years for males and 41.0 years for females, which is consistent with the Actuarial Society of South Africa’s estimates (males 36.3 years; females 42.4 years) [23]. Patients therefore receive up to 10 tests in their life-time. Each test costs $9.30, which includes the cost of the screening rapid HIV test kit, outpatient department counselor salaries and the cost of space [22]. The total undiscounted cost of the medical facility-based program over the lifetime of an HIV-negative patient is calculated to be $95.70. Over an entire lifetime, at an annual discount rate of 3% compounded monthly, the per-person cost HIV screening is $46.70. The total lifetime medical facility-based program test cost is incurred by each HIV-negative individual upon model entry. For the Budget Impact Analysis, in which we only consider costs over the first 2 years, each HIV-negative individual only incurs costs for medical facility-based HIV testing over the 2-year period.

**CEPAC-I Disease Model**

***Overview***

The CEPAC International Model is a computer-based, state-transition, Monte Carlo simulation model of the progression and outcomes of HIV disease in a hypothetical cohort of patients in resource-limited settings. The structure of the model has been described in previous publications [1,2,3,4,5,6,7,8]. Each individual patient’s clinical course is followed from the time of entry into the model until death. A running tally is maintained of all clinical events, the length of time spent in each health state, and the costs associated with each health state. Upon the patient’s death, summary statistics are recorded and a new patient enters the model. This process is repeated over a large number of patients, at which point overall performance measures such as life expectancy and average lifetime cost are computed. Statistical convergence can typically be achieved with overall cohort sizes of five to ten million. The HIV-infected cohort was fixed at one million patients for all of our analyses, resulting in overall cohort sizes > 9 million patients.

Individuals in both screening strategies experience the same HIV disease progression, response to treatment, engagement with care (after initial linkage) and mortality. Input data for the Disease Model have been described and published previously [3,4]. Inputs not mentioned in the main manuscript are summarized below and in Table S1.

***Natural history***

Monthly CD4 count declines are derived from the Multicenter AIDS Cohort Study [24]. The monthly probability of infection for mild and severe OIs, which has been described previously, were derived from a study in Cape Town for HIV-infected individuals not on ART, and from a study in Côte d’Ivoire for patients on ART [3,12,25]. All individuals, regardless of HIV status, face a risk of death each month from non-HIV related causes. Mortality risks due to non-HIV related causes are derived from data published by the WHO [26]. HIV-infected individuals have an additional mortality risk as a result of acute OIs (within 30 days of OI diagnosis), chronic HIV (history of OI or other HIV complication), and ART toxicity [12,25]. HIV-related mortality rates and incidence are derived from the CTAC cohort [12,25].

***Engagement with care***

While patients who are linked to care via the CEPAC Screening Model attend routine clinic visits, undergo HIV RNA and CD4 count monitoring and go on prophylaxis and ART regimens, some patients can be lost to follow up (LTFU). After being lost, the patients no longer undergo HIV RNA and CD4 count monitoring, or receive prophylaxis and ART. Each patient’s CD4 and viral load trajectory follow the same pattern as when they are taken off ART for other reasons. While LTFU, patients may still visit the clinic for OI treatment, and attend limited routine clinic visits. Among those who are lost some individuals may return to care.

Patients linked to care in both strategies are subject to a monthly probability of being lost to follow up. The probability is calculated using logistic regression and differs depending on whether or not the patient has started ART. The likelihood of individuals becoming lost to follow-up is dependent on ART adherence level and derived from a systematic review of ART programs in sub-Saharan Africa [27,28]. Patients on ART with an ART adherence level >95% have a 0.002 monthly probability of becoming lost to follow-up, and those with an ART adherence level <50% have a 0.01 monthly probability of becoming lost to follow-up [27]. Cohort study data suggest that LTFU rates before initiating ART tend to be significantly higher than LTFU rates among patients on ART [29,30,31]. To reflect this difference, we assume that all patients who have not yet initiated ART have a 0.01 monthly probability of becoming lost to follow-up.

Due to the dearth of data on the likelihood of individuals who are lost to follow-up returning to care, we assume that all patients have a 0.5 probability of return to care in the first month after developing an AIDS-defining OI, and that after their first year, lost patients have a 0.01 monthly probability of returning to care. Upon return to care we assume that all patients on suppressive ART continue the same regimen. Patients on first-line ART who are observed to be failing switch to second-line ART, while those previously on second-line ART remain on second-line ART. Patients with an unobserved failure continued on their previous ART regimen. Patients who were previously observed to be failing had a 0% probability of suppression upon returning to the same regimen. Patients who were previously suppressed have a 96% probability of suppression [32].

***Prophylaxis treatment and laboratory monitoring***

HIV-infected individuals in care with a CD4 count <500/µL are initiated on co-trimoxazole prophylaxis; prophylaxis efficacy is derived from data on a cohort in Côte d’Ivoire [12,33,34]. As per South Africa guidelines, patients in care receive laboratory CD4 counts every 6 months prior to ART initiation and annually thereafter [35,36]. HIV RNA tests are performed 6 months post-ART initiation, and annually thereafter [35,36]. In both strategies, patients in care receive routine laboratory CD4 counts (cost: $13.90) and routine HIV RNA count (cost: $69.50) [37].

***ART treatment***

As per South African guidelines, ART is initiated upon diagnosis of a severe AIDS-defining OI or TB, regardless of CD4 count, or with a CD4 count ≤350/µL [36,38]. Two ART regimens are available to individuals over the course of their lifetime [35]. Due to limited data on second-line efficacy, we assume the same rates of virologic suppression for first and second-line ART [39]. Patients on ART experience a reduction in HIV RNA and a CD4 count increase dependant on their ART adherence level. ART efficacy parameters are derived from a Côte d’Ivoire-based study and stratified by ART adherence [40]. Individuals experiencing virologic suppression are susceptible to treatment failure resulting in virologic rebound and CD4 decline. In the first 2 months of successful virologic suppression, individuals experience a 67/µL CD4 count increase, followed by a 3/µL increase per month until virologic failure [41].

**RESULTS**

The projected life expectancy of the HIV-negative population (from 33 years of age) was 35.7 years among males and 41.5 years among females, consistent with the Actuarial Society of South Africa’s 2008 estimates of 36.3 years among males and 42.4 years among females [23].

**Sensitivity Analyses**

Results reported in the text provide information regarding sensitivity analyses for the most influential input parameters. Herein, we provide details of one-way sensitivity analyses on all major inputs, as well as multi-way sensitivity analyses for parameters that did not have a meaningful impact on overall conclusions. Confidence intervals are not used in CEPAC model analyses because very large population sizes can be simulated such that confidence intervals are artificially narrow.

***One-way sensitivity analyses***

Figure S1 presents details on one-way sensitivity analyses on all key input parameters. As reported in the manuscript, results were most sensitive to variations in the prevalence of previously undiagnosed HIV, the medical facility-based program HIV testing frequency, and linkage to care among mobile unit testers.

***Prevalence and Mobile Unit Cost***

The HIV prevalence by province in South Africa varies from 5% in the Western Cape to 26% in KwaZulu-Natal [42]. We anticipate that provincial differences will also affect the cost to purchase, operate and maintain a mobile HIV testing unit. To evaluate the benefit of the mobile unit in different provinces we conducted two-way sensitivity analyses on the prevalence of previously undiagnosed HIV and cost of the mobile unit intervention purchase, operation and maintenance. For each province we evaluated the cost-effectiveness of the mobile unit at 0.5-2.0 times the cost of the mobile unit intervention purchase, operation and maintenance. The mobile unit intervention remained very cost-effective under all conditions (<$3,600/YLS). Table S2 summarizes the outcomes for the province with the highest prevalence of previously undiagnosed HIV, KwaZulu-Natal, and the province with the lowest prevalence of previously undiagnosed HIV, Western Cape Town, at double the base case mobile unit purchase, operation and maintenance cost.

***Test Acceptance and Linkage to Care***

To understand the maximum benefits that a screening program might provide, we examine the base case scenario under conditions of optimal mobile unit HIV test acceptance (100%), POC CD4 count acceptance (100%) and subsequent linkage to care (100%). Under these conditions the addition of the mobile unit intervention increased discounted life expectancy to 151.5 months for HIV-infected individuals and 251.1 months in the overall population. Compared to medical facility-based testing, the mobile unit intervention with optimal test acceptance and linkage to care had a cost-effectiveness ratio of $1,800/YLS.

In two-way sensitivity analysis, we then varied the test acceptance and linkage to care rates from 20%-100% in 20% increments (Figure S2). The mobile unit remained very cost-effective under all scenarios of test acceptance and linkage, but was no longer very cost-effective at a test acceptance rate of 30% and linkage to care rate of 20%.

***Loss to follow-up and return to care***

To evaluate the benefit of optimal retention in care we examined the base case scenario with no LTFU. With no loss from care the discounted HIV-infected population life expectancy increased to 148.2 months in the medical facility-based testing strategy and 159.8 in the mobile unit intervention strategy. The overall population life expectancy increased to 250.9 months in the medical facility-based testing strategy and 251.7 in the mobile unit intervention strategy with a resultant ICER of $2,000/YLS.

In two-way sensitivity analyses, we then varied the monthly probability of LTFU from 0.001-0.015 (base case 0.01) among patients with an ART adherence rate <50% and the monthly probability of return to care after one-year lost from 0.001-0.015 (base case 0.01). The mobile unit remained very cost-effective under all scenarios of LTFU and return and care. Table S3 summarizes outcomes for the scenario with the most favorable scenario (low LTFU of 0.001 and high return to care rate of 0.015) and the least favorable scenario (high LTFU of 0.015 and low return to care of 0.001).

***HIV transmissions averted***

Timely and effective ART reduces the risk of HIV transmission when patients have a suppressed viral load [43,44,45,46,47,48,49]. When compared to medical facility-based testing only, the mobile unit intervention resulted in more patients linking to care and initiating ART. In order to assess the additional public health benefits of mobile unit testing, we calculated the first-order HIV transmissions that would be averted with the addition of a mobile unit intervention to medical facility-based testing. In addition to the results summarized in the body of the manuscript, model projected outcomes included the number of patients in each different HIV RNA stratum per month. With these outcomes in both strategies, we used the probability of HIV transmissions among HIV discordant couples stratified by HIV RNA [50] to approximate the cumulative number of HIV transmissions over 5 years per 1,000 HIV-infected individuals. In the medical facility-based testing only strategy, we estimate 323 HIV transmissions per 1,000 HIV-infected individuals over 5 years, compared to 298 in the mobile unit strategy. Therefore, the addition of mobile HIV testing to medical facility-based testing is projected to avert approximately 25 more HIV transmissions per 1,000 HIV-infected individuals.

**Table S1. Additional base case input parameters for an analysis of a mobile HIV testing unit in Cape Town, South Africa**

| **Variable** | | | | | **Base Case** | **Ref.** |
| --- | --- | --- | --- | --- | --- | --- |
| Initial HIV RNA distribution (%) | | | | |  | [51] |
|  | >100,000 copies/mL | | | | 44 |  |
|  | 30,001-100,000 copies/mL | | | | 29 |  |
|  | 10,001-30,000 copies/mL | | | | 19 |  |
|  | 3,001-10,000 copies/mL | | | | 8 |  |
|  | ≤3,000 copies/mL | | | | 0 |  |
| **Natural history of disease** | | | | | |  |
| Mean monthly CD4 decline by HIV RNA level (cells/µL) | | | | | | [24] |
|  | | 30,000-100,000 copies/mL | | | 6.4 |  |
|  | | 10,001-30,000 copies/mL | | | 5.4 |  |
|  | | 3,001-10,000 copies/mL | | | 4.6 |  |
|  | | 501-3,000 copies/mL | | | 3.7 |  |
|  | | <500 copies/mL | | | 3.0 |  |
| Monthly risk of severe opportunistic disease* (%) | | | | |  | [12] |
|  | | | Bacterial | | 0.04-0.71 |  |
|  | | | Tuberculosis | | 0.16-1.96 |  |
|  | | | WHO stage 3-4 visceral diseases | | 0.04-1.52 |  |
|  | | | WHO stage 3-4 muco-cutaneous diseases | | 0.02-2.26 |  |
|  | | | Non-specific WHO stage 3-4 defining diseases | | 0.02-0.71 |  |
|  | | | | Other WHO stage 3-4 diseases | 0.20-1.67 |  |

**Table S1. Additional base case input parameters for an analysis of a mobile HIV testing unit in Cape Town, South Africa (continued)**

| **Variable** | | | | **Base Case** | **Ref.** |
| --- | --- | --- | --- | --- | --- |
| Monthly risk of mild opportunistic disease* (%) | | | |  | [12] |
|  | | Fungal | | 1.76-3.14 |  |
|  | | Other | | 2.33-2.67 |  |
| **HIV detection and linkage to care** | | | | | |
| *following presentation with severe OI* | | | | | |
| Probability of HIV detection and linkage  in month of acute infection (%) | | | | |  |
|  | | | Tuberculosis | 50 | Assumption |
|  | | | WHO stage diseases | 100 | Assumption |
| **Co-trimoxazole prophylaxis** (initiation at CD4 <500 cells/µL) | | | | |  |
| Efficacy (% reduction in probability of infection) | | | |  | [33] |
|  | | | Severe bacterial | 49.8 |  |
|  | | | Mild fungal§ | -46.4 |  |
|  | | | WHO stage 3-4 visceral diseases | 17.9 |  |
|  | | | Other WHO stage 3-4 diseases | 17.9 |  |
| **ART treatment** | | | |  |  |
| (1^st^ line TDF/3TC/EFV, 2^nd^ line AZT/3TC/LPVr) | | | | | [39,41] |
| Probability of HVL suppression at 6 months (%) (1^st^ and 2^nd^ line therapy) | | | | | [40] |
|  | Adherence >95% | | | 91 |  |
|  | Adherence <50% | | | 30 |  |

**Table S1. Additional base case input parameters for an analysis of a mobile HIV testing unit in Cape Town, South Africa (continued)**

| **Variable** | **Base Case** | **Ref.** |  |
| --- | --- | --- | --- |
| Overall mean (1^st^-line only) | 79 |  |  |
| Virologic failure rate per 100 person years after 6 months |  | [40] |  |
| Adherence >90% | 1.6 |  |  |
| Adherence <50% | 93.3 |  |  |

*Range indicated by CD4 count

§ The risk of mild fungal infection increased by 46.4% with co-trimoxazole prophylaxis

OI: Opportunistic Infection; SD: standard deviation; ART: antiretroviral therapy; TDF: tenofovir; 3TC: lamivudine; EFV: efavirenz; AZT: zidovudine; LPVr: ritonavir-boosted lopinavir

**Table S2. Two-way sensitivity analyses on the impact of prevalence of previously undiagnosed HIV and mobile unit purchase, operation and maintenance cost on model outcomes and cost-effectiveness of mobile unit HIV testing in Cape Town, South Africa**

| Total population outcomes | | Medical Facility-based | Mobile unit intervention |
| --- | --- | --- | --- |
| **KwaZulu-Natal (26% prevalence),** | | | |
| **2x mobile unit cost $1,043,200** | | | |
|  | Discounted life expectancy (months) | 225.5 | 227.7 |
|  | Discounted per-person costs ($) | 5,480 | 5,850 |
|  | Incremental cost-effectiveness ratio^*^ ($/YLS) | -- | 2,000 |
| **Western Cape (5% prevalence),** | | | |
| **2x mobile unit cost $1,043,200** | | | |
|  | Discounted life expectancy (months) | 251.9 | 252.3 |
|  | Discounted per-person costs ($) | 3,840 | 3,970 |
|  | Incremental cost-effectiveness ratio^*^ ($/YLS) | -- | 3,500 |

^*^Incremental cost-effectiveness ratios < 1xSouth African *per capita* gross domestic product ($8,200) considered very cost-effective as per WHO guidelines [52]. Costs in 2012 US$. Discounted at 3% per year. YLS: years of life saved

**Table S3. Model outcomes and cost-effectiveness of mobile unit HIV testing in Cape Town, South Africa under the most and least favorable LTFU and return to care conditions**

| Total population outcomes | | Medical Facility-based | Mobile unit intervention |
| --- | --- | --- | --- |
| **LTFU 0.0010 (monthly probability),** | | | |
| **return to care 0.0150 (monthly probability)** | | | |
|  | Discounted life expectancy (months) | 250.3 | 250.9 |
|  | Discounted per-person costs ($) | 4,030 | 4,150 |
|  | Incremental cost-effectiveness ratio^*^ ($/YLS) | -- | 2,200 |
| **LTFU 0.0150 (monthly probability),** | | | |
| **return to care 0.0010 (monthly probability)** | | | |
|  | Discounted life expectancy (months) | 249.5 | 250.0 |
|  | Discounted per-person costs ($) | 3,920 | 4,010 |
|  | Incremental cost-effectiveness ratio^*^ ($/YLS) | -- | 2,400 |

^*^Incremental cost-effectiveness ratios < 1xSouth African *per capita* gross domestic product ($8,200) considered very cost-effective as per WHO guidelines [52]. Costs in 2012 US$. Discounted at 3% per year. YLS: years of life saved

**LEGEND TO FIGURES**

**Figure S1.** One-way sensitivity analyses on the addition of mobile unit HIV testing to medical facility-based testing. This tornado diagram summarizes the results of multiple 1-way sensitivity analyses on the incremental cost-effectiveness of the addition of mobile unit HIV testing to medical facility-based testing in Cape Town, South Africa. The horizontal bars represent the incremental cost-effectiveness ratio (ICER) range as a result of variations in each single model parameter. The solid vertical line indicates the base case ICER ($2,400/LYS). The dashed vertical line indicates the South Africa *per capita* gross domestic product (GDP, $8,200). YLS: years of life saved; POC: point of care. (range; base case); SOC: standard of care.

**Figure S2.** Two-way sensitivity analyses on mobile unit test acceptance and linkage to care. This diagram shows the incremental cost-effectiveness of the addition of mobile unit HIV testing to medical facility-based testing under conditions of varied mobile unit test acceptance and linkage to care. Linkage to care is varied on the vertical axis and test acceptance is on the horizontal axis.

**LITERATURE CITED**

1. Freedberg KA, Losina E, Weinstein MC, Paltiel AD, Cohen CJ, et al. (2001) The cost effectiveness of combination antiretroviral therapy for HIV disease. N Engl J Med 344: 824-831.

2. Paltiel AD, Weinstein MC, Kimmel AD, Seage GR, 3rd, Losina E, et al. (2005) Expanded screening for HIV in the United States--an analysis of cost-effectiveness. N Engl J Med 352: 586-595.

3. Walensky RP, Wood R, Fofana MO, Martinson NA, Losina E, et al. (2011) The clinical impact and cost-effectiveness of routine, voluntary HIV screening in South Africa. J Acquir Immune Defic Syndr 56: 26-35.

4. Walensky RP, Wood R, Weinstein MC, Martinson NA, Losina E, et al. (2008) Scaling up antiretroviral therapy in South Africa: the impact of speed on survival. J Infect Dis 197: 1324-1332.

5. Ciaranello AL, Lockman S, Freedberg KA, Hughes M, Chu J, et al. (2011) First-line antiretroviral therapy after single-dose nevirapine exposure in South Africa: a cost-effectiveness analysis of the OCTANE trial. AIDS 25: 479-492.

6. Paltiel AD, Freedberg KA, Scott CA, Schackman BR, Losina E, et al. (2009) HIV preexposure prophylaxis in the United States: impact on lifetime infection risk, clinical outcomes, and cost-effectiveness. Clin Infect Dis 48: 806-815.

7. Freedberg KA, Kumarasamy N, Losina E, Cecelia AJ, Scott CA, et al. (2007) Clinical impact and cost-effectiveness of antiretroviral therapy in India: starting criteria and second-line therapy. AIDS 21: S117-128.

8. Walensky RP, Wolf LL, Wood R, Fofana MO, Freedberg KA, et al. (2009) When to start antiretroviral therapy in resource-limited settings. Ann Intern Med 151: 157-166.

9. Paltiel AD, Walensky RP, Schackman BR, Seage GR, 3rd, Mercincavage LM, et al. (2006) Expanded HIV screening in the United States: effect on clinical outcomes, HIV transmission, and costs. Ann Intern Med 145: 797-806.

10. Govindasamy D, Kranzer K, van Schaik N, Noubary F, Wood R, et al. (2013) Linkage to HIV, TB and non-communicable disease care from a mobile testing unit in Cape Town, South Africa. PLoS One 8: e80017.

11. Heunis JC, Wouters E, Norton WE, Engelbrecht MC, Kigozi NG, et al. (2011) Patient- and delivery-level factors related to acceptance of HIV counseling and testing services among tuberculosis patients in South Africa: a qualitative study with community health workers and program managers. Implement Sci 6: 27.

12. Holmes CB, Wood R, Badri M, Zilber S, Wang B, et al. (2006) CD4 decline and incidence of opportunistic infections in Cape Town, South Africa: implications for prophylaxis and treatment. J Acquir Immune Defic Syndr 42: 464-469.

13. Walensky RP, Goldie SJ, Sax PE, Weinstein MC, Paltiel AD, et al. (2002) Treatment for primary HIV infection: projecting outcomes of immediate, interrupted, or delayed therapy. J Acquir Immune Defic Syndr 31: 27-37.

14. Kranzer K, van Schaik N, Karmue U, Middelkoop K, Sebastian E, et al. (2011) High prevalence of self-reported undiagnosed HIV despite high coverage of HIV testing: a cross-sectional population based sero-survey in South Africa. PLoS One 6: e25244.

15. Bartlett JG, Gallant JE (2001) Medical Management of HIV Infection. Baltimore, MD: Johns Hopkins University, Division of Infectious Diseases.

16. Glencross DK, Coetzee LM, Faal M, Masango M, Stevens WS, et al. (2012) Performance evaluation of the Pima point-of-care CD4 analyser using capillary blood sampling in field tests in South Africa. J Int AIDS Soc 15: 3.

17. Govindasamy D, van Schaik N, Kranzer K, Wood R, Mathews C, et al. (2011) Linkage to HIV care from a mobile testing unit in South Africa by different CD4 count strata. J Acquir Immune Defic Syndr 58: 344-352.

18. Larson BA, Brennan A, McNamara L, Long L, Rosen S, et al. (2010) Lost opportunities to complete CD4+ lymphocyte testing among patients who tested positive for HIV in South Africa. Bull World Health Organ 88: 675-680.

19. Statistics South Africa: Mid-year population estimates 2011. P0302 ed. Pretoria: Statistics South Africa.

20. Noubary F, Hughes MD (2010) Assessing agreement in the timing of treatment initiation determined by repeated measurements of novel versus gold standard technologies with application to the monitoring of CD4 counts in HIV-infected patients. Stat Med 29: 1932-1946.

21. van Schaik N, Kranzer K, Wood R, Bekker LG (2010) Earlier HIV diagnosis--are mobile services the answer? S Afr Med J 100: 671-674.

22. Bassett IV, Giddy J, Nkera J, Wang B, Losina E, et al. (2007) Routine voluntary HIV testing in Durban, South Africa: the experience from an outpatient department. J Acquir Immune Defic Syndr 46: 181-186.

23. (2011) Actuarial Society of South Africa 2008 Estimates. Available: http://aids.actuarialsociety.org.za/ASSA2008-Model-3480.htm. Accessed 22 April 2013.

24. Mellors JW, Muñoz A, Giorgi JV, Margolick JB, Tassoni CJ, et al. (1997) Plasma viral load and CD4+ lymphocytes as prognostic markers of HIV-1 infection. Ann Intern Med 126: 946-954.

25. Losina E, Yazdanpanah Y, Deuffic-Burban S, Wang B, Wolf LL, et al. (2007) The independent effect of highly active antiretroviral therapy on severe opportunistic disease incidence and mortality in HIV-infected adults in Côte d'Ivoire. Antivir Ther 12: 543-551.

26. (2009) United Nations, Department of Economic and Social Affairs, Population Division, World Population Prospects: The 2008 Revision. New York.

27. Fox MP, Rosen S (2010) Patient retention in antiretroviral therapy programs up to three years on treatment in sub-Saharan Africa, 2007-2009: systematic review. Trop Med Int Health 1: 1-15.

28. Brinkhof MW, Pujades-Rodriguez M, Egger M (2009) Mortality of patients lost to follow-up in antiretroviral treatment programmes in resource-limited settings: systematic review and meta-analysis. PLoS One 4: e5790.

29. Larson BA, Brennan A, McNamara L, Long L, Rosen S, et al. (2010) Early loss to follow up after enrolment in pre-ART care at a large public clinic in Johannesburg, South Africa. Trop Med Int Health 1: 43-47.

30. Hassan AS, Fielding KL, Thuo NM, Nabwera HM, Sanders EJ, et al. (2012) Early loss to follow-up of recently diagnosed HIV-infected adults from routine pre-ART care in a rural district hospital in Kenya: a cohort study. Trop Med Int Health 17: 82-93.

31. Kranzer K, Govindasamy D, Ford N, Johnston V, Lawn SD (2012) Quantifying and addressing losses along the continuum of care for people living with HIV infection in sub-Saharan Africa: a systematic review. J Int AIDS Soc 15: 17383.

32. Danel C, Moh R, Chaix ML, Gabillard D, Gnokoro J, et al. (2009) Two-months-off, four-months-on antiretroviral regimen increases the risk of resistance, compared with continuous therapy: a randomized trial involving West African adults. J Infect Dis 199: 66-76.

33. Yazdanpanah Y, Losina E, Anglaret X, Goldie SJ, Walensky RP, et al. (2005) Clinical impact and cost-effectiveness of co-trimoxazole prophylaxis in patients with HIV/AIDS in Côte d'Ivoire: a trial-based analysis. AIDS 19: 1299-1308.

34. Goldie SJ, Kaplan JE, Losina E, Weinstein MC, Paltiel AD, et al. (2002) Prophylaxis for human immunodeficiency virus-related *Pneumocystis carinii* pneumonia: using simulation modeling to inform clinical guidelines. Arch Intern Med 162: 921-928.

35. SANAC (2010) South Africa National Department of Health Clinical Guidelines for the Management of HIV & AIDS in Adults and Adolescents. Available: http://www.who.int/hiv/pub/guidelines/south_africa_art.pdf. Accessed 23 July 2013.

36. Matsoso MP (April 14, 2012) Letter Re: Accelerating Access to ART Services and Uptake Pretoria: South Africa Department of Health. Available: http://www.tbonline.info/media/uploads/documents/changes_to_art_guidelines.pdf. Accessed 23 July 2013.

37. Cleary S, Okorafor O, Chitha W, Boulle A, Jikwana S (2005) South African Health Review: Chapter 5 - Financing Antiretroviral Treatment and Primary Health Care Services. Durban: Health Systems Trust. Available: http://www.healthlink.org.za/uploads/files/sahr05_chapter5.pdf. Accessed 23 July 2013.

38. SANAC (2010) The South African Antiretroviral Treatment Guidelines. South Africa Department of Health. Available: http://www.uj.ac.za/EN/CorporateServices/ioha/Documentation/Documents/ART%20Guideline.pdf. Accessed 23 July 2013.

39. Hammond R, Harry TC (2008) Efficacy of antiretroviral therapy in Africa: effect on immunological and virological outcome measures -- a meta-analysis. Int J STD AIDS 19: 291-296.

40. Messou E, Chaix ML, Gabillard D, Minga A, Losina E, et al. (2012) Association between medication possession ratio, virologic failure and drug resistance in HIV-1 infected adults on antiretroviral therapy in Côte d'Ivoire. J Acquir Immune Defic Syndr 56: 356-364.

41. Tuboi SH, Brinkhof MW, Egger M, Stone RA, Braitstein P, et al. (2007) Discordant responses to potent antiretroviral treatment in previously naive HIV-1-infected adults initiating treatment in resource-constrained countries: the antiretroviral therapy in low-income countries (ART-LINC) collaboration. J Acquir Immune Defic Syndr 45: 52-59.

42. Shisana O, Rehle T, Simbayi L, Parker W, Jooste S, et al. (2009) South African National HIV Prevalence, Incidence, Behaviour and Communication Survey, 2008. Cape Town: HSRC Press.

43. Seyler C, Anglaret X, Dakoury-Dogbo N, Messou E, Toure S, et al. (2003) Medium-term survival, morbidity and immunovirological evolution in HIV-infected adults receiving antiretroviral therapy, Abidjan, Côte d'Ivoire. Antivir Ther 8: 385-393.

44. Ray M, Logan R, Sterne JA, Hernandez-Diaz S, Robins JM, et al. (2010) The effect of combined antiretroviral therapy on the overall mortality of HIV-infected individuals. AIDS 24: 123-137.

45. Ivers LC, Kendrick D, Doucette K (2005) Efficacy of antiretroviral therapy programs in resource-poor settings: a meta-analysis of the published literature. Clin Infect Dis 41: 217-224.

46. Venkatesh KK, Lurie MN, Mayer KH (2012) How HIV treatment could result in effective prevention. Future Virol 5: 405-415.

47. Granich RM, Gilks CF, Dye C, De Cock KM, Williams BG (2009) Universal voluntary HIV testing with immediate antiretroviral therapy as a strategy for elimination of HIV transmission: a mathematical model. Lancet 373: 48-57.

48. Dieffenbach CW, Fauci AS (2009) Universal voluntary testing and treatment for prevention of HIV transmission. JAMA 301: 2380-2382.

49. Cohen MS, Chen YQ, McCauley M, Gamble T, Hosseinipour MC, et al. (2011) Prevention of HIV-1 infection with early antiretroviral therapy. N Engl J Med 365: 493-505.

50. Gray RH, Wawer MJ, Brookmeyer R, Sewankambo NK, Serwadda D, et al. (2001) Probability of HIV-1 transmission per coital act in monogamous, heterosexual, HIV-1-discordant couples in Rakai, Uganda. Lancet 357: 1149-1153.

51. Lawn SD, Badri M, Wood R (2005) Tuberculosis among HIV-infected patients receiving HAART: long term incidence and risk factors in a South African cohort. AIDS 19: 2109-2116.

52. WHO (2012) CHOosing Interventions that are Cost Effective (WHO-CHOICE): Cost-effectiveness thresholds. Geneva: World Health Organization. Available: http://www.who.int/choice/costs/CER_thresholds/en/index.html. Accessed 23 July 2013.
