# Supplementary figures and images for: Mobile HIV Screening in Cape Town, South Africa: Clinical Impact, Cost and Cost-Effectiveness
Source: PLoS One. 2014 Jan 22;9(1):e85197. doi: 10.1371/journal.pone.0085197 (PMC3898963; doi:10.1371/journal.pone.0085197)

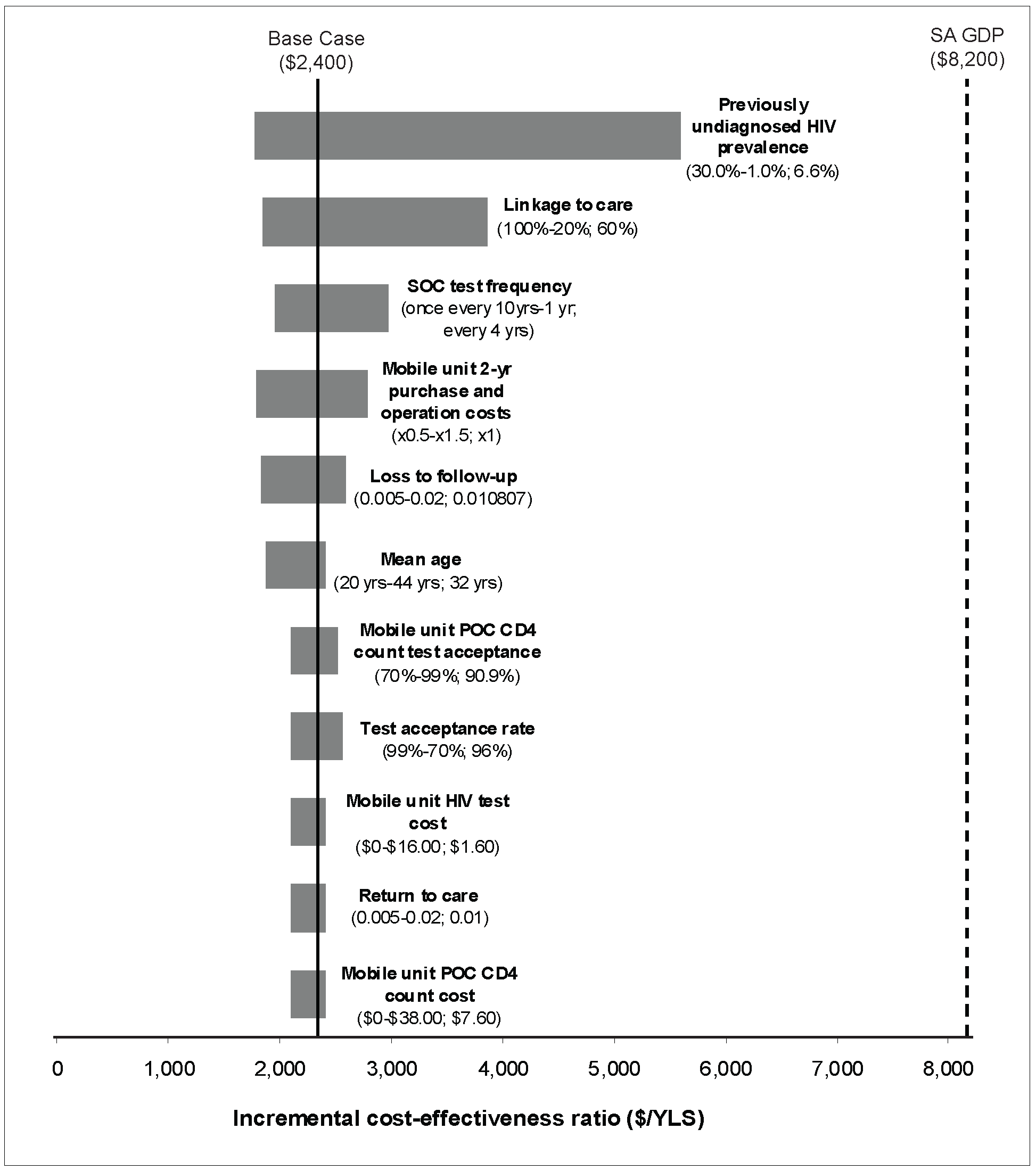

Supplement: Figure S1 — One-way sensitivity analyses on the addition of mobile unit HIV testing to medical facility-based testing. This tornado diagram summarizes the results of multiple 1-way sensitivity analyses on the incremental cost-effectiveness of the addition of mobile unit HIV testing to medical facility-based testing in Cape Town, South Africa. The horizontal bars represent the incremental cost-effectiveness ratio (ICER) range as a result of variations in each single model parameter. The solid vertical line indicates the base case ICER ($2,400/LYS). The dashed vertical line indicates the South Africa per capita gross domestic product (GDP, $8,200). YLS: years of life saved; POC: point of care. (range; base case); SOC: standard of care. (TIFF) [file pone.0085197.s002.tiff]

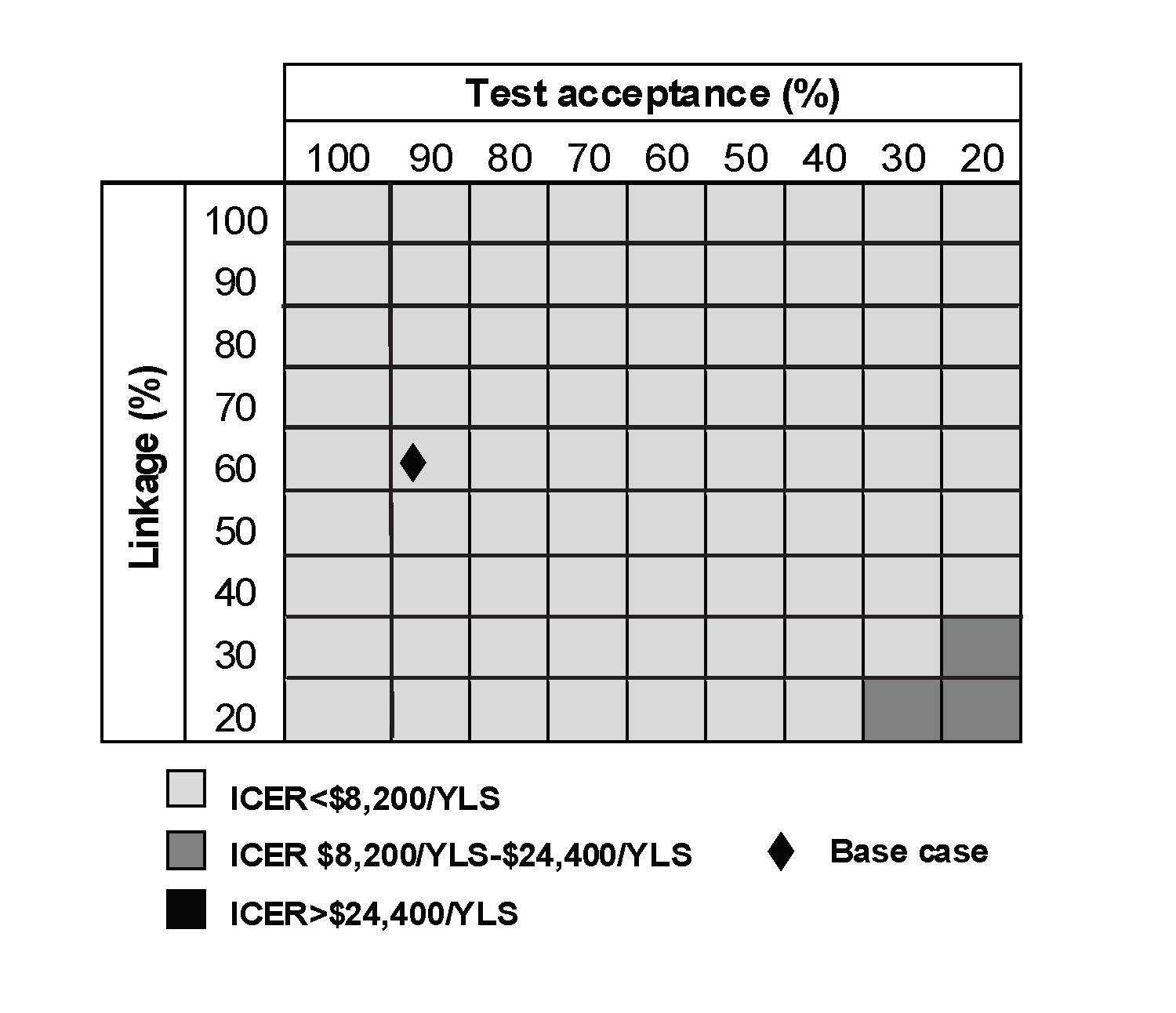

Supplement: Figure S2 — Two-way sensitivity analyses on mobile unit test acceptance and linkage to care. This diagram shows the incremental cost-effectiveness of the addition of mobile unit HIV testing to medical facility-based testing under conditions of varied mobile unit test acceptance and linkage to care. Linkage to care is varied on the vertical axis and test acceptance is on the horizontal axis. (TIF) [file pone.0085197.s003.tif]
